# Association of Eosinophilic Inflammation with *FKBP51* Expression in Sputum Cells in Asthma

**DOI:** 10.1371/journal.pone.0065284

**Published:** 2013-06-06

**Authors:** Tomoko Tajiri, Hisako Matsumoto, Akio Niimi, Isao Ito, Tsuyoshi Oguma, Hitoshi Nakaji, Hideki Inoue, Toshiyuki Iwata, Tadao Nagasaki, Yoshihiro Kanemitsu, Guergana Petrova, Michiaki Mishima

**Affiliations:** 1 Department of Respiratory Medicine, Graduate School of Medicine, Kyoto University, Kyoto, Japan; 2 Division of Respiratory Medicine, Department of Medical Oncology and Immunology, Nagoya City University School of Medical Sciences, Aichi, Japan; 3 Department of Respiratory Medicine, Japanese Red Cross Wakayama Medical Center, Wakayama, Japan; Research Center Borstel, Germany

## Abstract

**Background:**

Airway eosinophilia is a predictor of steroid responsiveness in steroid-naïve asthma. However, the relationship between airway eosinophilia and the expression of FK506-binding protein 51 (FKBP51), a glucocorticoid receptor co-chaperone that plays a role in steroid insensitivity in asthma, remains unknown.

**Objective:**

To evaluate the relationship between eosinophilic inflammation and *FKBP51* expression in sputum cells in asthma.

**Methods:**

The *FKBP51* mRNA levels in sputum cells from steroid-naïve patients with asthma (n = 31) and stable asthmatic patients on inhaled corticosteroid (ICS) (n = 28) were cross-sectionally examined using real-time PCR. Associations between *FKBP51* levels and clinical indices were analyzed.

**Results:**

In steroid-naïve patients, the *FKBP51* levels were negatively correlated with eosinophil proportions in blood (r = −0.52) and sputum (r = −0.57), and exhaled nitric oxide levels (r = −0.42) (all p<0.05). No such associations were observed in patients on ICS. In steroid-naïve patients, improvement in forced expiratory volume in one second after ICS initiation was correlated with baseline eosinophil proportions in blood (r = 0.74) and sputum (r = 0.76) and negatively correlated with *FKBP51* levels (r = −0.73) (all p<0.0001) (n = 20). Lastly, the *FKBP51* levels were the lowest in steroid-naïve asthmatic patients, followed by mild to moderate persistent asthmatic patients on ICS, and the highest in severe persistent asthmatic patients on ICS (p<0.0001).

**Conclusions:**

Lower *FKBP51* expression in sputum cells may reflect eosinophilic inflammation and glucocorticoid responsiveness in steroid-naïve asthmatic patients.

## Introduction

Asthma is a chronic inflammatory disorder of the airways in which eosinophils, Th2 cells, and Th2-type cytokines play a role [1]. Glucocorticosteroid (GC), an established key treatment in asthma, efficiently reduces cytokine production and induces apoptosis of eosinophils [2] and Th2 cells *via* GC receptor α (GRα). Thus, eosinophilia in asthma is responsive to GC; steroid-naïve asthmatic patients with blood [3], [4] or sputum [3], [5] eosinophilia show greater improvement in lung function after GC treatment than patients without eosinophilia.

FK506-binding protein 51 (FKBP51) is a co-chaperone of GR and is expressed in various tissues and cell types [6]. FKBP51 is induced by the auto-regulatory process of GC-activated GR [7], modulates GRα activity, and plays a role in GC insensitivity, which may be a homeostatic reaction for regulating the effects of GC, similar to the reduction in GR number following GC treatment [8]. In steroid-naïve patients with asthma, lower expression of *FKBP51* mRNA in airway epithelial cells [9] and in peripheral blood mononuclear cells [10] is correlated with greater improvement in lung function after GC treatment, suggesting that low expression of *FKBP51* may be a mechanism underlying the GC sensitivity. However, a potential association between *FKBP51* expression and airway inflammatory cells, in particular eosinophils, has not been reported.

In this study, we examined *FKBP51* expression in induced sputum cells in patients with asthma to test the hypothesis that the level of *FKBP51* expression is down-regulated in eosinophilic inflammation in steroid-naïve asthma, and that this down-regulation disappears in patients on inhaled corticosteroid (ICS) treatment.

## Patients and Methods

### Patients

Newly referred steroid-naïve patients with asthma and stable patients with asthma who were treated with ICS at the Asthma Clinic in the Kyoto University Hospital were enrolled. Asthma was defined according to the American Thoracic Society criteria [11]. The asthmatic patients on ICS were stable, and they had been free of exacerbations for 4 weeks or more. Patients who had smoked within the previous 6 months or who had failed sputum induction were excluded. The disease severity of asthma on ICS was classified into four categories: intermittent, mild persistent, moderate persistent, and severe persistent, according to the Global Initiative for Asthma guidelines, as revised in 2002 [12], after determining the minimum medication necessary to maintain control.

Healthy participants who had not smoked within the previous 6 months were recruited from our hospital staff.

The study protocol (UMIN000005106) was approved by the Ethics Committee of Kyoto University, and written informed consent was obtained from all participants.

## Methods

In this study, patients with asthma cross-sectionally underwent the following examination: fractional exhaled nitric oxide (FeNO) levels, pulmonary function test, sputum induction, and blood test. In steroid-naïve patients with asthma, a follow-up pulmonary function test was also performed after they were treated with the minimum ICS dose needed to maintain control.

Peripheral blood was obtained from healthy controls, and eosinophils were purified as described below.

### Measurement of FeNO Levels

FeNO levels at an expiratory flow rate of 50 ml/s were measured with a chemiluminescence analyzer (NOA 280; Sievers, Boulder, Colorado, USA) [13] according to current guidelines [14].

### Pulmonary Function Test

After FeNO measurements, pre-bronchodilator forced expiratory volume in one second (FEV_1_) was measured using a Chest Graph HI-701 spirometer (Chest, Tokyo, Japan). Spirometry was performed according to the standards of the American Thoracic Society and the European Respiratory Society [15]. For steroid-naïve patients with asthma, follow-up FEV_1_ was also measured. Changes in FEV_1_ were calculated as 100 × (FEV_1_ at the 2^nd^ measurement − FEV_1_ at baseline)/FEV_1_ at baseline.

### Sputum Induction, RNA Isolation from Sputum Cells, Real-time PCR, and Immunostaining for *FKBP51* Expression in Sputum Cells

Sputum induction and processing were performed as described previously [16]. Adequate plugs of sputum were separated from saliva, stored at 4°C, and processed within 2 hours. The sputum plugs were treated with 0.1% dithiothreitol (Sputasol, Oxoid Ltd., Hampshire, UK) followed by Dulbecco’s phosphate-buffered saline (PBS). After centrifugation, supernatants were removed, and cell pellets were re-suspended in PBS. Sputum cells were mounted on slides by cytocentrifugation, air-dried, and fixed in acetone/methanol (75∶25). Cell differentials were determined by counting at least 400 non-squamous cells on a slide that was stained with the May-Grünwald-Giemsa method. The remaining slides were stored at −20°C and used for immunostaining as described below.

Total RNA was extracted from the remaining cells using an RNeasy Mini kit (Qiagen, Osaka, Japan). cDNA was synthesized, and real-time PCR was performed using the ABI Prism 7300 sequence detection system (Applied Biosystems, Tokyo, Japan) with SYBR green (Qiagen). The relative quantity of *FKBP51* mRNA expression levels was normalized to the mRNA expression levels of *β_2_ microglobulin* (*β_2_MG*) in the same sample. The specific primer sets used were forward 5′-CCAAAGCTGTTGAATGCTGTGA-3′ and reverse 5′-CAAACTCGTTCATGAGCAGCTG-3′ for *FKBP51*, and forward 5′-TGTCTTTCAGCAAGGACTGGTC-3′ and reverse 5′-CAAACCTCCATGATGCTGC-3′ for *β_2_MG* [17].

We also evaluated FKBP51 protein expression with immunocytochemistry in sputum cells. For double immunostaining, previously prepared samples on the slides were first blocked with CAS Block (Invitrogen Corp., Carlsbad, California, USA) and then incubated with either rabbit anti-human FKBP51 (4 µg/ml) (Santa Cruz Biotechnology, Santa Cruz, California, USA) or rabbit IgG (Santa Cruz Biotechnology) at the same concentration, and either mouse anti-human major basic protein (MBP; Chemicon, Temecula, California, USA) or mouse IgG (Sigma-Aldrich, Tokyo, Japan). After rinsing in PBS, samples were incubated with Alexa Fluor 488 donkey anti-rabbit IgG (Invitrogen) and Alexa Fluor 546 goat anti-mouse IgG (Invitrogen). Samples were viewed with a fluorescence microscope. Positive staining was green for the FKBP51 antigen and red for the MBP antigen.

### Purification of Blood Eosinophils, Real-time PCR, and Immunostaining for *FKBP51* Expression in Purified Blood Cells

Peripheral blood was obtained from healthy controls, and *FKBP51* mRNA expression in purified eosinophils, neutrophils and mononuclear cells [18] was examined. Briefly, granulocytes were isolated from mononuclear cells by sedimentation with 2% dextran, followed by centrifugation on 1.103 and 1.085 Percoll (GE Healthcare, Uppsala, Sweden) density gradients as modified from previous reports [18], [19]. After lysis of red blood cells with 0.2% and 1.6% saline, eosinophils and neutrophils were purified by negative and positive selection, respectively, using anti-CD16 immunomagnetic beads and the mini-MACS system (Miltenyi Biotec, Bergish Gladbach, Germany).

Total RNA was extracted from individual pools of purified eosinophils, neutrophils, and mononuclear cells, and the levels of *FKBP51* mRNA expression normalized to *β_2_MG* were determined as described above.

We also evaluated FKBP51 protein expression with immunocytochemistry in individual pools of purified eosinophils, neutrophils, and mononuclear cells. Purified blood cells were smeared on slides, air-dried, fixed in acetone/methanol (75∶25), and immunostained as described above.

### RNA Quality Assessment

RNA quality was determined using the Experion Automated Electrophoresis System (BIO-RAD, Tokyo, Japan) according to the manufacturer’s instructions. RNA integrity was expressed as the RNA quality indicator (RQI), which ranged from 1 (degraded) to 10 (intact) [20]. Samples were categorized as having poor RNA integrity if 1≤ RQI ≤4, as having moderate RNA integrity if 4< RQI ≤7, and as having high RNA integrity if 7< RQI ≤10, according to the manufacturer’s instructions.

### Statistical Analysis

JMP system version 6 (SAS Institute Japan; Tokyo, Japan) was used. Data are expressed as the mean ± SD or median (range). Eosinophil proportions in blood and sputum, *FKBP51* mRNA levels normalized to *β_2_MG* in sputum cells, ICS doses, and changes in FEV_1_ were log-transformed to achieve normal distributions. For parametric data, Pearson correlation coefficients were used to analyze the relationships among the data, and unpaired t-test was used to compare two groups. For comparison of three groups, the chi-squared test, Kruskal-Wallis test, or analysis of variance was used, where appropriate. A p value of <0.05 was considered statistically significant.

## Results

### Patient Characteristics

The patients’ characteristics are shown in Table 1. A total of six patients with mild to moderate persistent asthma and 22 patients with severe persistent asthma were included in the group of asthmatic patients on ICS. Severe persistent asthmatics on ICS showed the longest disease duration and the lowest FEV_1_ among the three patient groups (Table 1). Sputum and blood eosinophil proportions did not differ among the three groups. One steroid-naïve patient with asthma was unable to undergo FeNO measurement because of time constraints. The average RQI of our sputum samples was 8.5±1.9. The RQI was independent of the cell type; no association was found between RQI and proportion of cell type (neutrophils (r = −0.06, p = 0.67), mononuclear cells (r = 0.15, p = 0.29), or eosinophils (r = 0.09, p = 0.54)).

**Table 1:** Patients’ characteristics.

	Steroid-naïve patientswith asthma	Mild to moderatepersistent asthmaticson ICS*	Severe persistentasthmatics on ICS	p-value†
Patients, number	31	6	22	
Gender, male/female	16/15	3/3	11/11	0.99†
Age, years	53±17	57±23	57±16	0.48†
Smoking history, ex/never	7/24	1/5	10/12	0.15†
Disease duration, years	4±6	10±12	17±19	0.0008¶
Atopic status‡, yes/no	22/9	4/2	18/4	0.60†
Doses of ICS§, µg daily	–	283±134	1214±696	0.0001#
FEV_1_, % predicted	100±26	101±27	83±25	0.01†
Exhaled nitric oxide levels, ppb	35±31	42±20	37±32	0.50†
Blood eosinophils, %	4±4	4±6	4±4	0.94#
Sputum eosinophils, %	11±22	5±5	7±10	0.87#
Serum IgE, IU/ml	83 (5–1106)	86 (9–220)	185 (5–1800)	0.19¶

Values are given as means ± SD or medians (range).

* included four patients with mild and two with moderate persistent asthma.

† with the χ^2^ test or analysis of variance.

‡ Patients were considered atopic when they were positive for one or more serum allergen-specific IgE antibodies against house dust, Japanese cedar pollen, mixed gramineae pollen, mixed weed pollen, mixed mold, cat dander, dog dander, and *Trichophyton rubrum*.

§ Equivalent to fluticasone propionate.

¶ by Kruskal Wallis test,

# by unpaired t-test or analysis of variance after data were log-transformed.

Abbreviations: ICS, inhaled corticosteroid; FEV_1_; forced expiratory volume in one second.

### *FKBP51* Expression in Induced Sputum Cells from Steroid-naïve Asthmatic Patients

**Figure 1:**
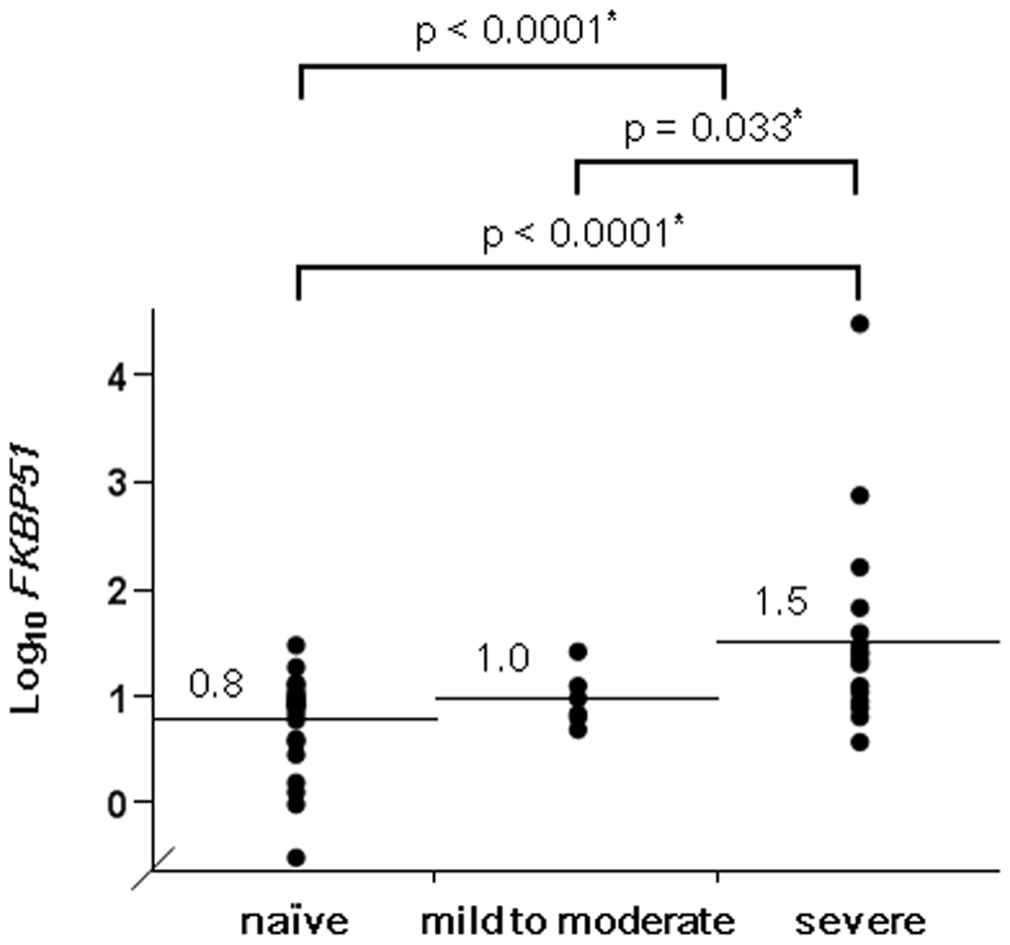
*FKBP51* levels in induced sputum cells in patients with asthma. *FKBP51* mRNA levels normalized to *β_2_ microglobulin* mRNA levels in induced sputum cells became progressively higher from steroid-naïve asthmatic patients (naïve, n = 31), to mild to moderate asthmatic patients on inhaled corticosteroid (mild to moderate, n = 6), and then to severe persistent asthmatic patients on inhaled corticosteroid (severe, n = 22) (p<0.0001 by the Kruskal-Wallis test). *Significant by the Wilcoxon rank-sum test. Values and bars represent means.

**Figure 2:**
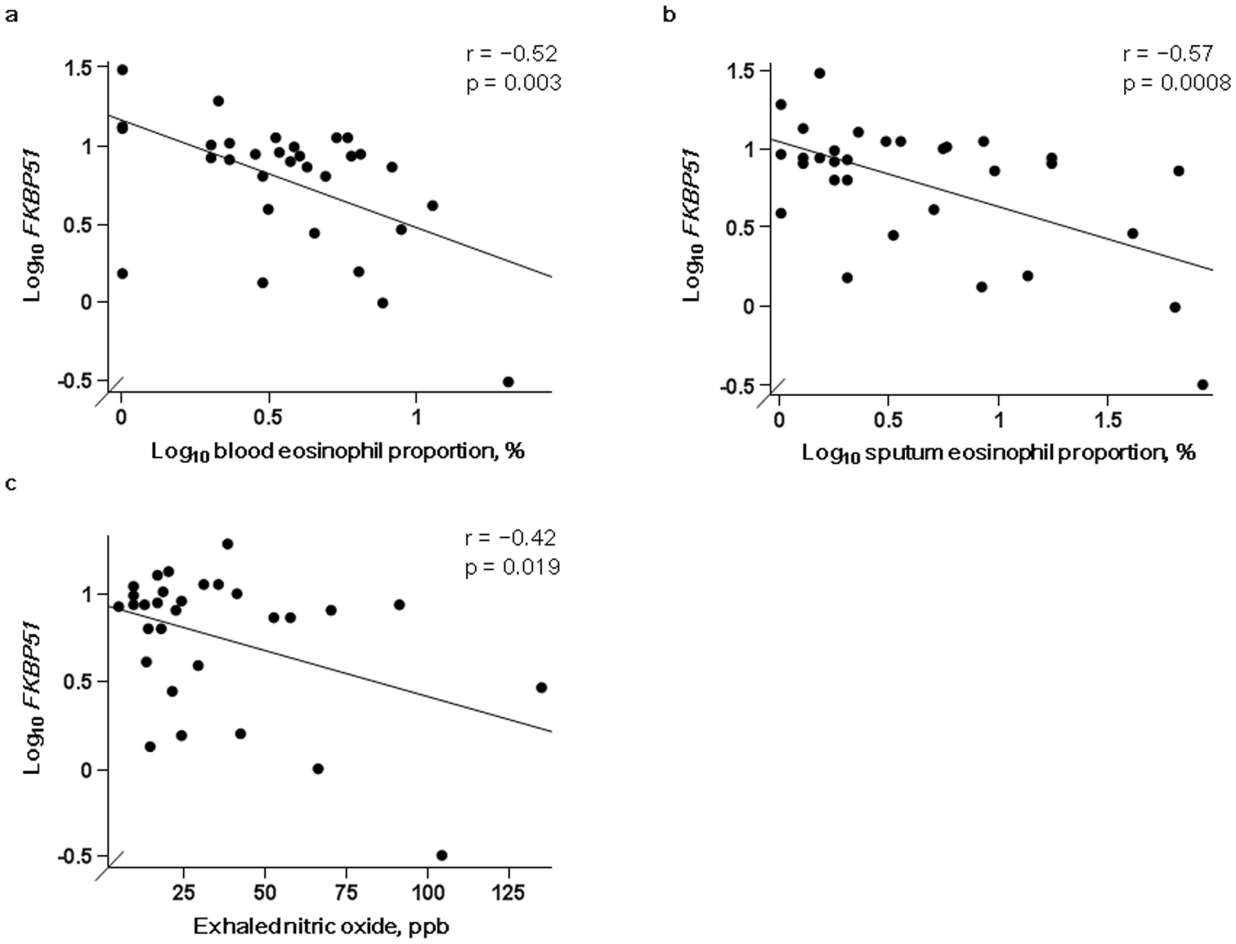
Associations between *FKBP51* levels and eosinophilic inflammation in steroid-naïve patients with asthma. Associations between *FKBP51* mRNA levels normalized to *β_2_ microglobulin* mRNA levels and a) blood and b) sputum eosinophil proportions (n = 31 each) and c) exhaled nitric oxide levels (n = 30) in steroid-naïve patients with asthma.

The level of *FKBP51* expression in induced sputum cells in steroid-naïve patients with asthma was significantly lower than that in patients on ICS (p<0.0001) (Fig. 1). In steroid-naïve patients with asthma, *FKBP51* expression was significantly inversely correlated with eosinophil proportions in blood (r = −0.52, p = 0.003) and sputum (r = −0.57, p = 0.0008) (Fig. 2a, b), and with FeNO levels (r = −0.42, p = 0.019) (Fig. 2c). The significant correlation between *FKBP51* expression and sputum eosinophil proportions remained even after the right most and lowest outlier in Fig. 2b was excluded from the analysis (r = −0.45, p = 0.013). When using a second order regression equation for *FKBP51* expression levels and sputum eosinophil proportions in steroid-naïve patients with asthma, *FKBP51* expression in a sputum non-eosinophil cell (i.e., neutrophil, mononuclear cell, or lymphocyte) was estimated to be 6.1 times higher than that in a sputum eosinophil. We applied 100 to “sputum eosinophil proportion” in the equation of “log_10_ *FKBP51* (expression normalized to *β_2_MG*) = 0.948 − 0.246 × (log_10_ sputum eosinophil proportion) − 0.101 × (log_10_ sputum eosinophil proportion − 0.246)^2″^ to estimate *FKBP51* expression in a sputum eosinophil, whereas 0.01 was used to estimate *FKBP51* expression in a non-eosinophil cell.

**Figure 3:**
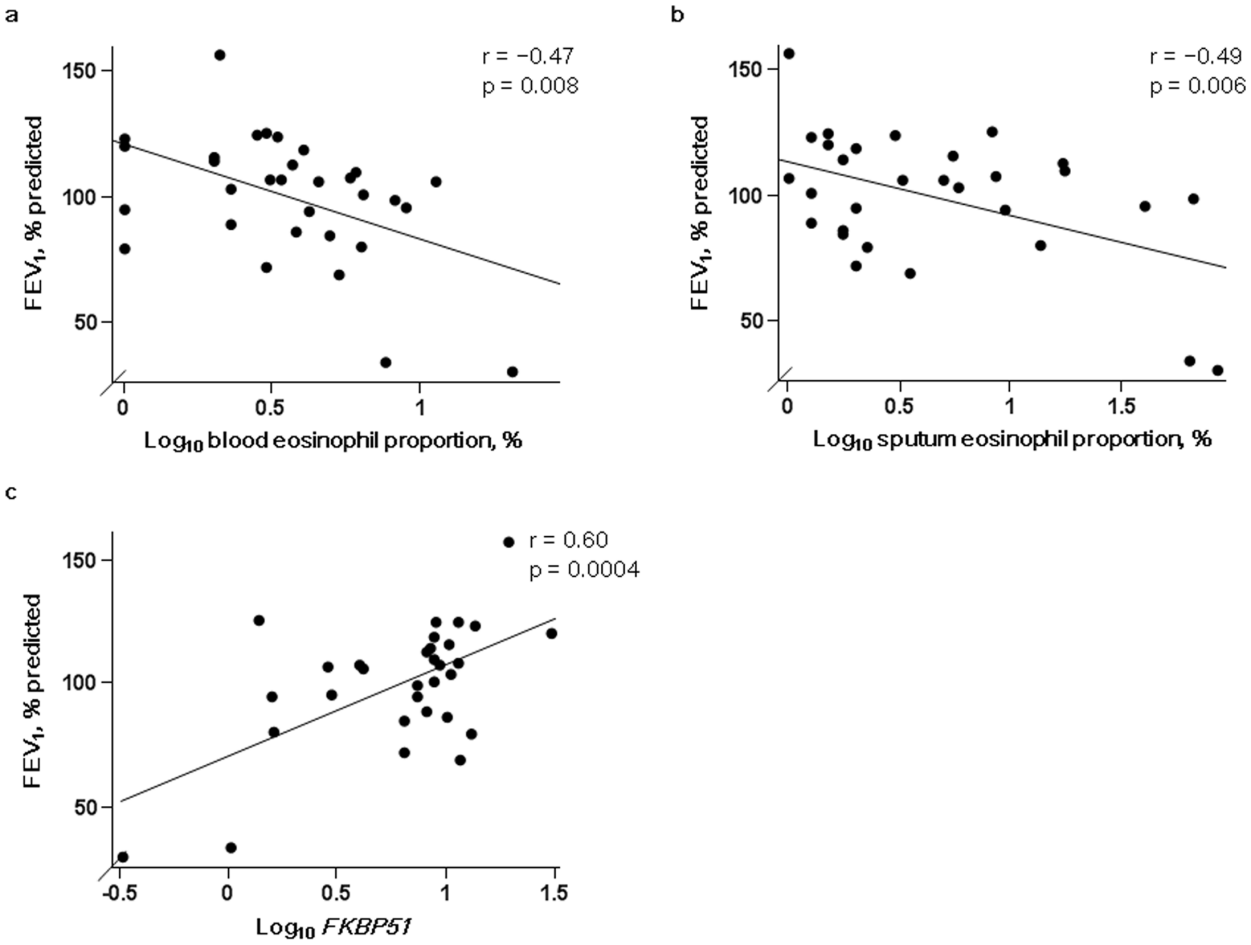
Associations between pretreatment FEV_1_ and eosinophilic inflammation and *FKBP51* levels in steroid-naïve patients with asthma. Associations between pretreatment FEV_1_ (% predicted) and a) blood and b) sputum eosinophil proportions and c) *FKBP51* mRNA levels normalized to *β_2_ microglobulin* mRNA levels in induced sputum cells in steroid-naïve patients with asthma (n = 31). Abbreviation: FEV_1_, forced expiratory volume in one second.

FEV_1_ (% predicted) was significantly negatively correlated with eosinophil proportions in blood (r = −0.47, p = 0.008) (Fig. 3a) and sputum (r = −0.49, p = 0.006) (Fig. 3b), and was positively correlated with *FKBP51* expression (r = 0.60, p = 0.0004) (Fig. 3c). The significant correlation between *FKBP51* expression and FEV_1_ (% predicted) remained after the left most outlier in Fig. 3c was excluded from the analysis (r = 0.44, p = 0.015). No significant associations were seen between *FKBP51* expression and sputum neutrophil or lymphocyte proportions or other clinical indices including sex, age, smoking history, disease duration, and atopic status (data not shown). Epithelial cell counts were too low for analysis (0.3±0.5%).

**Figure 4:**
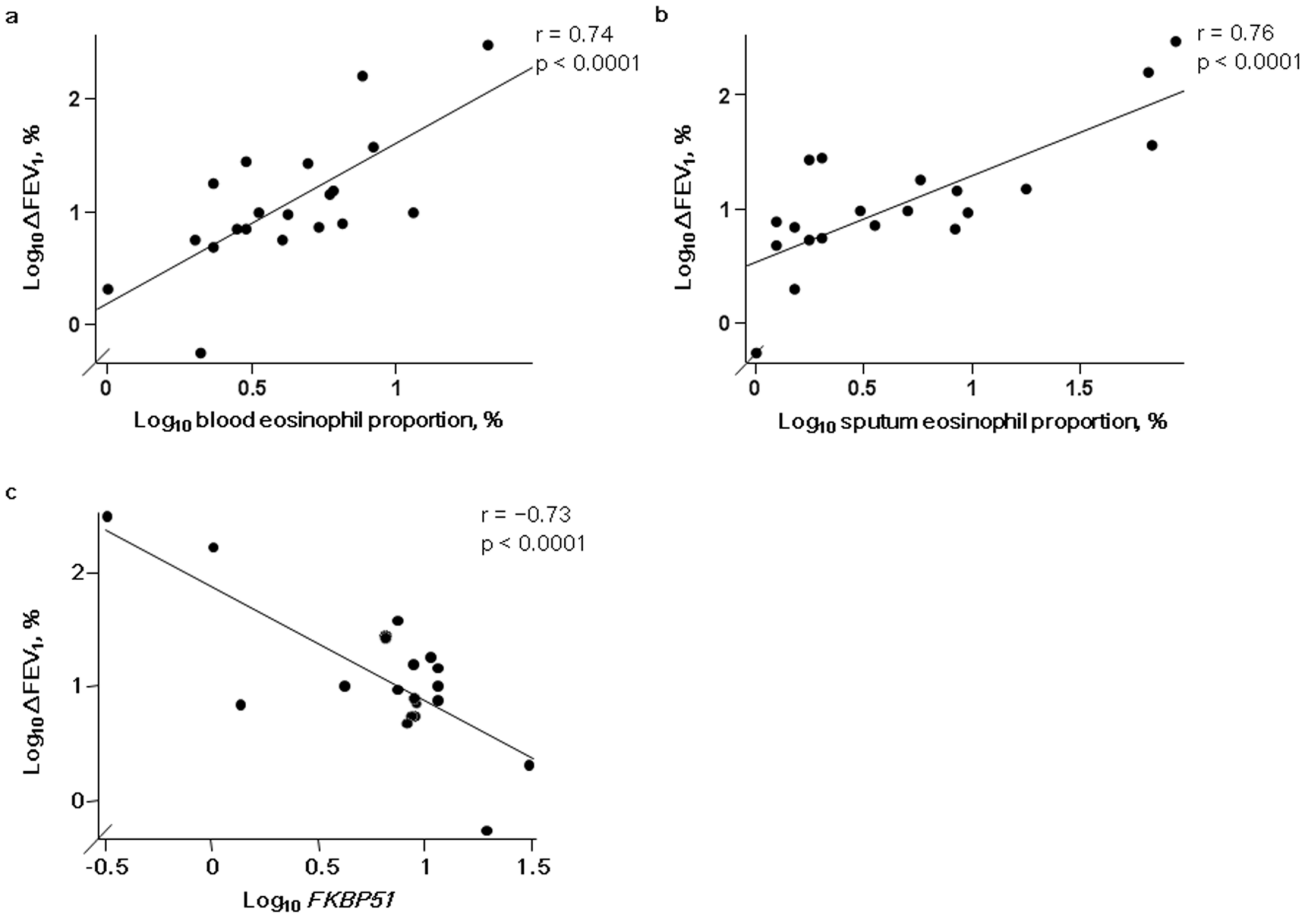
Associations between changes in FEV_1_ after ICS initiation and pretreatment eosinophilic inflammation and *FKBP51* levels. Associations between changes in FEV_1_ after ICS initiation and pretreatment a) blood and b) sputum eosinophil proportions and c) *FKBP51* mRNA levels normalized to *β_2_ microglobulin* mRNA levels in steroid-naïve patients with asthma (n = 20). Abbreviation: FEV_1_, forced expiratory volume in one second; ICS, inhaled corticosteroid.

A total of 20 steroid-naïve asthmatic patients were followed up at our hospital. They underwent a 2^nd^ pulmonary function test 11.4±3.8 months later when they were on minimum ICS doses to maintain control (399±241 µg daily equivalent to fluticasone propionate). Changes in FEV_1_ (24.7±73.7%) from baseline to the 2^nd^ measurement were significantly positively correlated with baseline eosinophil proportions in blood (r = 0.74, p<0.0001) (Fig. 4a) and sputum (r = 0.76, p<0.0001) (Fig. 4b), and were negatively correlated with *FKBP51* expression (r = −0.73, p<0.0001) (Fig. 4c). We did not observe any differences in sex, age, baseline FEV_1_ (% predicted), eosinophil proportions in blood and sputum, or *FKBP51* mRNA levels between the 20 patients and the 11 patients who were lost to follow-up.

**Figure 5:**
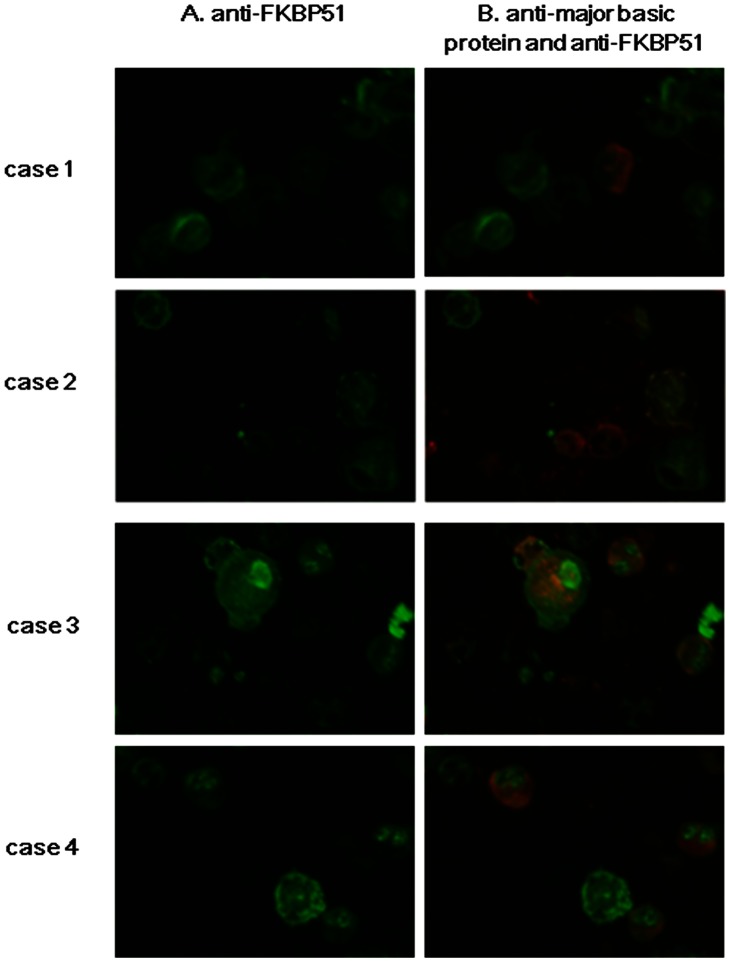
Representative images of immunostaining of sputum cells from asthmatic patients. Case 1 (68-year-old male) and case 2 (72-year-old female) were steroid-naïve patients. Case 3 (79-year-old male) and case 4 (55-year-old female) were patients with severe persistent asthma on high-dose inhaled corticosteroid. Column A: staining with anti-FKBP51 antibody, column B: merged image of staining with anti-major basic protein antibody (MBP) and anti-FKBP51 antibody. Red indicates MBP, and green indicates FKBP51.

Using immunocytochemistry, we observed that FKBP51 expression was qualitatively weaker in sputum eosinophils than in sputum neutrophils and mononuclear cells in steroid-naïve asthmatic patients (Fig. 5, cases 1,2).

### *FKBP51* Expression in Induced Sputum Cells in Asthmatic Patients on ICS

In asthmatic patients on ICS, the level of *FKBP51* expression in patients with severe persistent asthma (n = 22) was significantly higher than that in patients with mild to moderate persistent asthma (n = 6) (p = 0.033) (Fig. 1). ICS doses were not significantly positively correlated with *FKBP51* expression (r = 0.28, p = 0.15) (n = 28).

In contrast to steroid-naïve patients with asthma, no significant associations were observed between *FKBP51* expression and eosinophil proportions in blood (r = 0.27, p = 0.17) and sputum (r = 0.28, p = 0.15) or FeNO levels (r = 0.23, p = 0.23) in stable asthmatic patients on ICS. Associations were also not observed between *FKBP51* expression and sputum neutrophil or lymphocyte proportions, FEV_1_ (% predicted), or other clinical indices (data not shown). Epithelial cell counts were too low for analysis (0.4±0.7%).

In asthmatic patients on ICS, immunostaining for FKBP51 in sputum eosinophils, particularly in the nucleus, was comparable to or stronger than that in neutrophils and mononuclear cells in severe persistent asthmatic patients on ICS (Fig. 5, cases 3, 4).

### *FKBP51* mRNA and Protein Expression in Purified Blood Eosinophils and Non-eosinophils

Eosinophils, neutrophils, and mononuclear cells were purified from the peripheral blood of 11 healthy controls (6 males and 5 females, 34.7±4.3 years old). The *FKBP51* mRNA levels in purified mononuclear cells were significantly higher than those in purified eosinophils, but not different from those in purified neutrophils. When neutrophils and mononuclear cells were analyzed together as non-eosinophils, *FKBP51* mRNA levels in non-eosinophils were 6.0±13.8 fold higher than those in eosinophils (p = 0.026).

**Figure 6:**
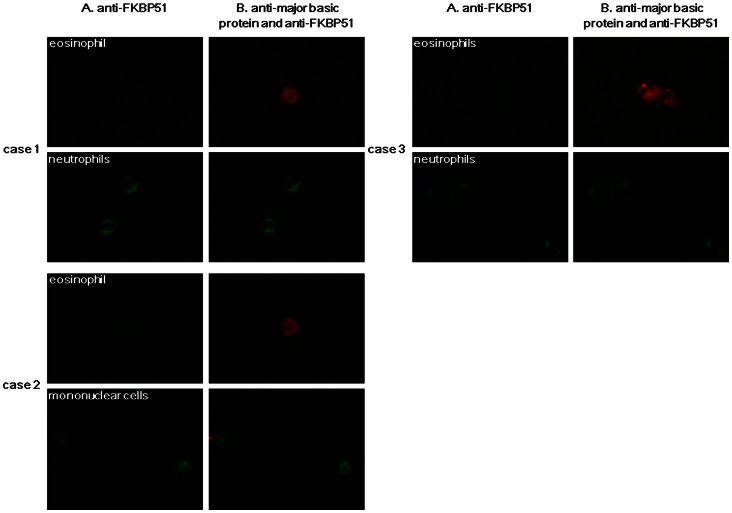
Representative images of immunostaining of purified blood eosinophils and non-eosinophils from healthy controls. Case 1 (46-year-old female), case 2 (36-year-old female), case 3 (35-year-old male). Column A: staining with anti-FKBP51 antibody, column B: merged image of staining with anti-major basic protein antibody (MBP) and anti-FKBP51 antibody. Red indicates MBP, and green indicates FKBP51.

Immunostaining for FKBP51 in purified eosinophils was also weaker than that in neutrophils or mononuclear cells (Fig. 6).

## Discussion

To the best of our knowledge, this is the first study that clarifies the associations between the level of *FKBP51* mRNA expression in induced sputum cells and clinical indices in patients with asthma, in particular, patients with eosinophilic inflammation. We showed that the level of *FKBP51* expression in induced sputum cells 1) was significantly inversely correlated with eosinophilic inflammation and positively correlated with improvement in FEV_1_ with ICS treatment in steroid-naïve patients with asthma and 2) became progressively higher from steroid-naïve asthmatic patients, to mild to moderate persistent asthmatic patients on ICS, and then to severe persistent asthmatic patients on ICS. No correlation of eosinophilic inflammation to *FKBP51* expression in induced sputum cells was observed in patients on ICS.

FKBP51 is a co-chaperone of GR. It was originally discovered as a member of the progesterone receptor complex [21] and was then described in 1999 as playing a major role in steroid resistance in squirrel monkeys with high circulating levels of GC [22], [23]. In previous studies using cultured squirrel monkey lymphocytes and human lymphocytes, *FKBP51* mRNA was induced by GC [24], and its overexpression was thought to inhibit GRα signaling by reducing the binding affinity of GC to GRα [22], [25], impairing nuclear translocation of GRα [26] and promoting nuclear translocation of GRβ [27].

In steroid-naïve asthmatic patients, the level of *FKBP51* expression in induced sputum cells was inversely correlated with the proportions of blood and sputum eosinophils, suggesting that the level of *FKBP51* expression in eosinophilic inflammation was lower than that in non-eosinophilic inflammation under steroid-naïve conditions. Lower *FKBP51* expression in eosinophilic airway inflammation may be advantageous for GC signaling *via* GRα and may accelerate eosinophil apoptosis [2], [28]. In an earlier report, lower baseline FEV_1_ in patients with eosinophilic inflammation was a strong predictor of GC responsiveness [4]. In our study, eosinophilic inflammation and lower *FKBP51* expression were associated with lower baseline FEV_1_ (% predicted) and greater improvement in FEV_1_ after ICS treatment. Collectively, lower *FKBP51* may be one of the mechanisms underlying the relationship between eosinophilia with lower baseline FEV_1_ and GC responsiveness in steroid-naïve asthmatic patients.

The current findings imply that the level of *FKBP51* expression in sputum eosinophils may be lower than that in sputum neutrophils and mononuclear cells. Indeed, immunostaining of sputum cells revealed a weaker FKBP51 expression in eosinophils than that in neutrophils and mononuclear cells in steroid-naïve asthmatic patients. To confirm these findings, we purified eosinophils from neutrophils and mononuclear cells using blood samples obtained from healthy controls because purification of sputum eosinophils by separation from non-eosinophils was technically difficult. Using purified blood cells, we first observed that *FKBP51* expression in eosinophils was significantly lower than that in non-eosinophils. Moreover, the ratio of *FKBP51* expression in eosinophils to *FKBP51* expression in non-eosinophils in blood was comparable to the estimated ratio in sputum cells. Taken altogether, the findings in blood cells may support the findings that lower *FKBP51* expression in sputum cells reflects eosinophilic inflammation in steroid-naïve patients with asthma. Nonetheless, for the differences in *FKBP51* expression levels between sputum cells from steroid naïve patients and those from patients on ICS, which is mentioned below, we should consider the possibility that these differences may reflect a mean change between the patient groups studied and not simply reflect changes at the cellular level because we did not purify sputum cell populations in this study.

In contrast to the steroid-naïve group, negative associations between *FKBP51* expression and eosinophilic inflammation were not observed in patients with asthma who were treated with ICS. The level of *FKBP51* expression in severe persistent asthmatics on ICS was significantly higher than that in mild to moderate persistent asthmatics on ICS and in steroid-naïve patients. The highest expression of *FKBP51* in our patients with severe persistent asthma on ICS is consistent with the findings in earlier reports that high expression of *FKBP51* in steroid-naïve conditions is associated with insensitivity to GC treatment [9], [10] and reduced GC-mediated inhibition of interleukin-13 signaling [7]. Meanwhile, the treatment conditions in our study and in earlier studies were different, and the high level of *FKBP51* in severe persistent asthmatic patients on ICS in our study is thought to be mostly induced by high doses of ICS. Despite this, overexpression of *FKBP51* may be involved in the pathogenesis of severe persistent asthmatic patients, including steroid insensitivity.

Our study has several limitations. First, we did not examine the level of *FKBP51* expression and its function in purified eosinophils and other cells in sputum. This was because sputum eosinophil samples (8.9±17.0%) (n = 59) were contaminated with other cell types; neutrophils (63.3±22.6%), mononuclear cells (25.8±20.3%), lymphocytes (1.8±1.6%), and epithelial cells (0.4±0.6%), and purification of sputum eosinophils by separation from non-eosinophils was technically difficult. Instead, we performed double immunostaining for sputum cells and examined FKBP51 expression in blood eosinophils and neutrophils or mononuclear cells. Second, *FKBP51* expression levels in severe persistent asthmatics in the steroid-naïve condition are unknown because reducing ICS to examine changes in *FKBP51* expression in severe persistent asthmatics is ethically difficult. A longitudinal study with a larger sample size is needed to determine the *FKBP51* expression levels in severe persistent asthmatics in the steroid-naïve condition. In addition, we need to examine the actual FKBP51 function in response to GC, including the acceleration of nuclear translocation of GRβ in sputum cells. This may be achieved by knocking down *FKBP51* expression using siRNA in sputum cells such as sputum-derived macrophages [29]. One strong point of our study is that we validated the quality of the RNA that was extracted from induced sputum cells. Immediate processing of sputum samples (within 2 hours) may have resulted in the satisfactory results in RNA quality.

In conclusion, we demonstrated for the first time that lower *FKBP51* expression in induced sputum cells may reflect eosinophilic inflammation and may underlie the mechanism of GC sensitivity in eosinophilic inflammation in the steroid-naïve condition. Longitudinal studies are necessary to further clarify the clinical significance of overexpression of *FKBP51* in patients on steroids.
